# [^11^C]CHIBA-1001 as a Novel PET Ligand for α7 Nicotinic Receptors in the Brain: A PET Study in Conscious Monkeys

**DOI:** 10.1371/journal.pone.0003231

**Published:** 2008-09-18

**Authors:** Kenji Hashimoto, Shingo Nishiyama, Hiroyuki Ohba, Masaaki Matsuo, Tatsuhiko Kobashi, Makoto Takahagi, Masaomi Iyo, Takeru Kitashoji, Hideo Tsukada

**Affiliations:** 1 Division of Clinical Neuroscience, Chiba University Center for Forensic Mental Health, Chiba, Japan; 2 Division of Medical Treatment and Rehabilitation, Chiba University Center for Forensic Mental Health, Chiba, Japan; 3 PET Center, Central Research Laboratory, Hamamatsu Photonics K. K., Hamamatsu, Shizuoka, Japan; 4 Nard Institute, Ltd., Amagasaki, Hyogo, Japan; 5 Department of Psychiatry, Chiba University Graduate School of Medicine, Chiba, Japan; University of Auckland, New Zealand

## Abstract

**Background:**

The α7 nicotinic acetylcholine receptors (nAChRs) play an important role in the pathophysiology of neuropsychiatric diseases such as schizophrenia and Alzheimer's disease. However, there are currently no suitable positron emission tomography (PET) radioligands for imaging α7 nAChRs in the intact human brain. Here we report the novel PET radioligand [^11^C]CHIBA-1001 for *in vivo* imaging of α7 nAChRs in the non-human primate brain.

**Methodology/Principal Findings:**

A receptor binding assay showed that CHIBA-1001 was a highly selective ligand at α7 nAChRs. Using conscious monkeys, we found that the distribution of radioactivity in the monkey brain after intravenous administration of [^11^C]CHIBA-1001 was consistent with the regional distribution of α7 nAChRs in the monkey brain. The distribution of radioactivity in the brain regions after intravenous administration of [^11^C]CHIBA-1001 was blocked by pretreatment with the selective α7 nAChR agonist SSR180711 (5.0 mg/kg). However, the distribution of [^11^C]CHIBA-1001 was not altered by pretreatment with the selective α4β2 nAChR agonist A85380 (1.0 mg/kg). Interestingly, the binding of [^11^C]CHIBA-1001 in the frontal cortex of the monkey brain was significantly decreased by subchronic administration of the N-methyl-D-aspartate (NMDA) receptor antagonist phencyclidine (0.3 mg/kg, twice a day for 13 days); which is a non-human primate model of schizophrenia.

**Conclusions/Significance:**

The present findings suggest that [^11^C]CHIBA-1001 could be a novel useful PET ligand for *in vivo* study of the receptor occupancy and pathophysiology of α7 nAChRs in the intact brain of patients with neuropsychiatric diseases such as schizophrenia and Alzheimer's disease.

## Introduction

The most of neuronal nicotinic acetylcholine receptors (nAChRs) are ligand-gated ion channels composed of α and β subunits that assemble to form pentamers with a variety of physiological and pharmacological properties. Two major subtypes exist in the brain, those comprised of α4β2 and those comprised of α7 subunits [1], [2]. The former contribute >90% of the high affinity binding sites for nicotine in the rat brain, and the low affinity binding sites (α7 subunits) for nicotine are recognized by their nanomolar affinity for α-bungarotoxin. Several lines of evidence suggest that α7 nAChRs play a role in the pathophysiology of neuropsychiatric diseases such as schizophrenia, Alzheimer's disease, anxiety, depression, and drug addiction, and that α7 nAChRs are the most attractive therapeutic targets for these diseases [3]–[11]. Studies using postmortem human brain samples have demonstrated alterations in the levels of α7 nAChRs in the brains of patients with schizophrenia [12], [13] and Alzheimer's disease [14]–[16]. It is thus of great interest to examine whether α7 nAChRs are altered in the living brain of patients with neuropsychiatric diseases such as schizophrenia and Alzheimer's disease. It is also of interest to measure the receptor occupancy of potential therapeutic α7 nAChR drugs in the intact human brain.

**Figure 1:**
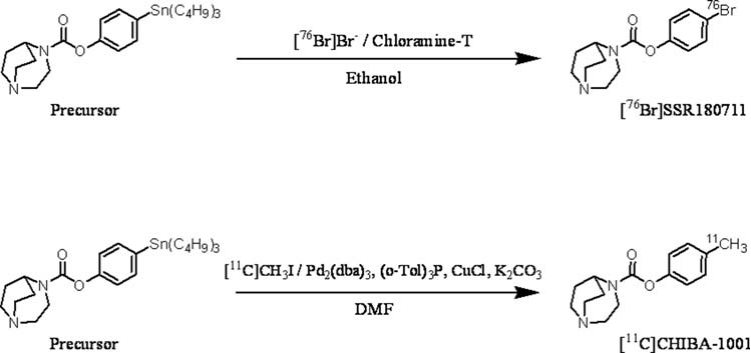
Synthesis of [^76^Br[SSR180711 and [^11^C]CHIBA-1001.

The distribution, density, and activity of receptors in the living brain can be visualized noninvasively by radioligands labeled for positron emission tomography (PET), and the receptor binding can be quantified by appropriate tracer kinetic models, which can be modified and simplified for particular applications [17]–[19]. The PET ligands ([^11^C]nicotine and 2-[^18^F]fluoro-A85380) for α4β2 nAChRs have been used in clinical studies [20]–[22]. However, there have been no clinical studies using PET ligands for α7 nAChRs in the human brain. Therefore, it is very important to develop a safe PET ligand for quantification of α7 nAChRs in the human brain. Very recently, researchers at Sanofi-Aventis developed the novel selective α7 nAChR agonist SSR180711 (4-bromophenyl 1,4-diazabicyclo(3.2.2) nonane-4-carboxylate)(Figure 1) [23], [24], which is under clinical study.

Here, we developed two novel PET ligands, [^76^Br]SSR180711 and [^11^C]CHIBA-1001, for *in vivo* imaging of α7 nAChRs in the human brain. Using conscious monkeys, we evaluated the two PET ligands for *in vivo* imaging of α7 nAChRs in the non-human primate brain. Furthermore, we evaluated the usefulness of [^11^C]CHIBA-1001 in a non-human primate model of schizophrenia.

## Results

### Receptor affinity and specificity

SSR180711 displaced specific binding of [^3^H]α-bungarotoxin to the rat and human α7 nAChRs with K_i_ values of 22 and 14 nM, respectively [23], and SSR180711 (10 µM) was found to be devoid of activity (inhibition lower than 50%) for a 100 standard receptor binding profile [23]. In our assay, the IC_50_ values of SSR180711 and CHIBA-1001 for [^125^I]α-bungarotoxin (0.5 nM) binding to the rat brain homogenates were 24.9 and 45.8 nM, respectively. Furthermore, CHIBA-1001 (1 µM) was found to be devoid of activity (inhibition lower than 50%) for a 28 standard receptor binding profile (See Supplemental Table S1 and S2).

### Synthesis of [^76^Br]SSR180711 and [^11^C]CHIBA1001

[^76^Br]SSR180711 and [^11^C]CHIBA-1001 were synthesized by bromination and methylation of the precursor, respectively (Figure 1). The radiochemical purity and specific activity of [^76^Br]SSR180711 were approximately 100% and 8.11±1.65 GBq/µmol (mean±SD of 9 experiments), respectively. The radiochemical yields and yields of [^76^Br]SSR180711 were 16.7±6.14% and 0.21±0.09 GBq (mean±SD of 9 experiments), respectively. The radiochemical purity and specific activity of [^11^C]CHIBA-1001 were 98.6±1.68% (mean±SD of 12 experiments) and 343.7±36.1 GBq/µmol (mean±SD of 12 experiments), respectively. The radiochemical yields and yields of [^11^C]CHIBA-1001 were 9.49±1.45% and 1.88±0.33 GBq (mean±SD of 12 experiments), respectively.

### Conscious monkey PET studies

**Figure 2:**
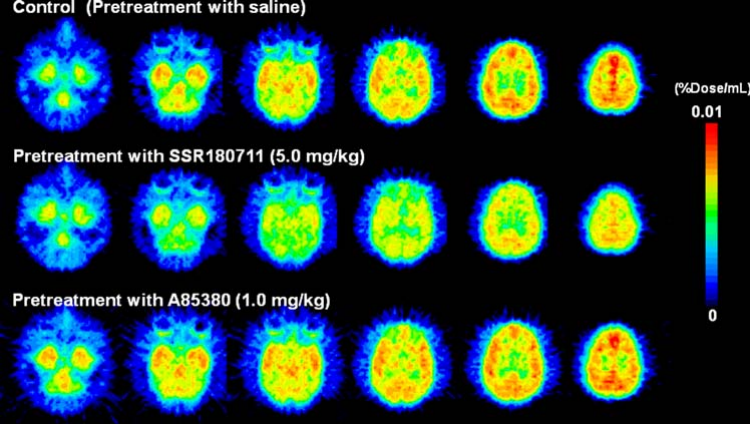
Representative PET images in the brains of a rhesus monkey after intravenous administration of [^76^Br]SSR180711. Upper: Control monkey (saline pre-treated). Middle: Pretreatment with SSR180711 (5.0 mg/kg, 30 min before). Lower: Pretreatment with A85380 (1.0 mg/kg, 30 min before)

**Figure 3 pone-0003231-g003:**
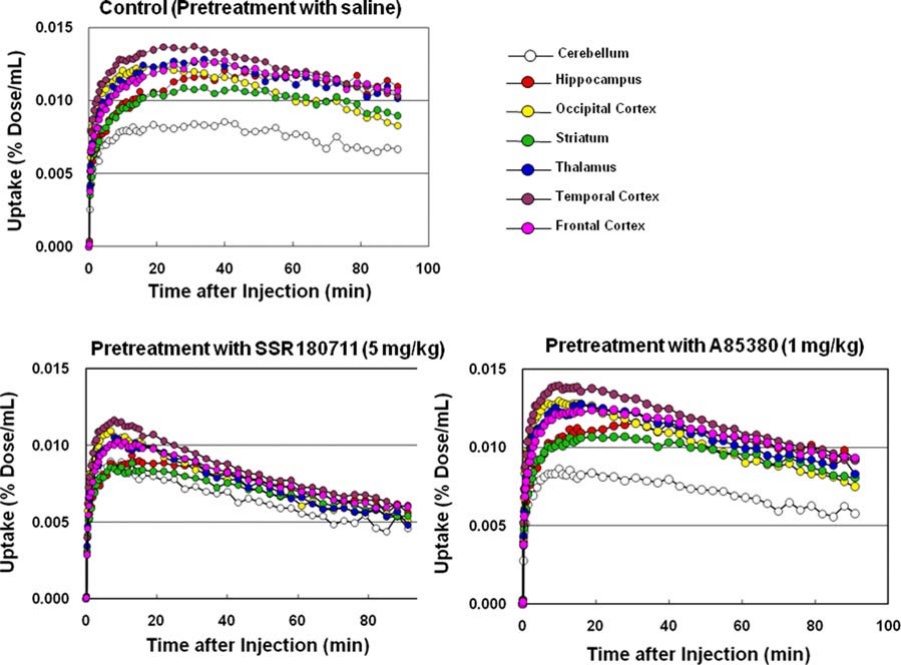
Representative time-activity curves of radioactivity (expressed as % Dose/mL) in several brain regions of a rhesus monkey after intravenous administration of [^76^Br[SSR180711 in control (saline pre-treated) monkey, SSR180711 (5.0 mg/kg, 30 min before)-pretreated monkey, and A85380 (1.0 mg/kg, 30 min before)-pretreated monkey.

**Figure 4:**
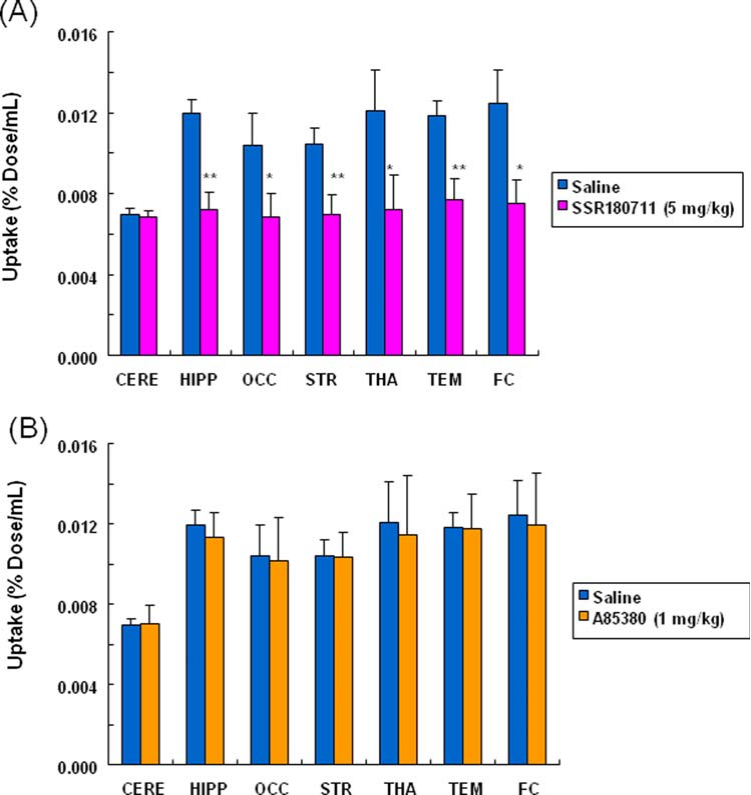
Effects of SSR180711 and A85380 on the uptake of the radioactivity in the monkey brain after intravenous administration of [^76^Br[SSR180711. (A): Uptake values (expressed as % Dose/mL) of [^76^Br[SSR180711 in several brain regions under control (saline pre-treated) group (during 70–91 min post-injection) and SSR180711 (5.0 mg/kg, 30 min before) treated groups. Data were the mean±S.D. of three monkeys. *p<0.05, **p<0.01 as compared to control group (Paired t-test). (B): Uptake values (expressed as % Dose/mL) of [^76^Br[SSR180711 in several brain regions under control (saline pre-treated) group (during 70–91 min post-injection) and A85380 (1.0 mg/kg, 30 min before) treated groups. Data were the mean±S.D. of three monkeys. CERE: cerebellum, HIPP: hippocampus, OCC: occipital cortex, STR: striatum, THA: thalamus, TEM: temporal cortex, FC: frontal cortex

Baseline PET scans showed rapid brain penetration and accumulation of [^76^Br]SSR180711 in the monkey brain (Figures 2–4). The peak time of radioactivity in the hippocampus was about 60 min after administration of the radioligand. Furthermore, the peak time of radioactivity in the other brain regions (occipital cortex, temporal cortex, frontal cortex, striatum, thalamus, and cerebellum) was about 30–40 min after administration of the radioligand. The distribution of radioactivity in the brain regions after administration of the radioligand was consistent with the distribution of α7 nAChRs in the monkey brain [25]–[27]. Uptake of radioactivity in the brain regions after intravenous administration of [^76^Br]SSR180711 was significantly decreased by pretreatment with the α7 nAChR agonist SSR180711 (5.0 mg/kg, i.v., 30 min)(Figures 2–4). Uptake of radioactivity (during 70–91 min) in the brain regions except the cerebellum (low receptor density) after intravenous administration of [^76^Br]SSR180711 was significantly decreased by pretreatment with the α7 nAChR agonist SSR180711 (5.0 mg/kg, i.v., 30 min)(Figures 4A). However, the distribution of radioactivity in the brain regions after intravenous administration of [^76^Br]SSR180711 was not altered by pretreatment with the selective α4β2 nAChR agonist A85380 (1.0 mg/kg, i.v., 30 min)[28], [29](Figures 2, 3 and 4B).

**Figure 5:**
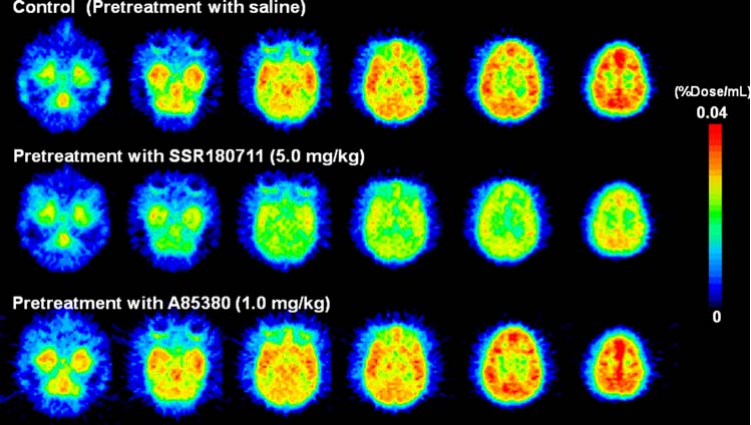
Representative PET images in the brains of a rhesus monkey after intravenous administration of [^11^C]CHIBA-1001. Upper: Control monkey (saline pre-treated). Middle: Pretreatment with SSR180711 (5.0 mg/kg, 30 min before). Lower: Pretreatment with A85380 (1.0 mg/kg, 30 min before)

**Figure 6 pone-0003231-g006:**
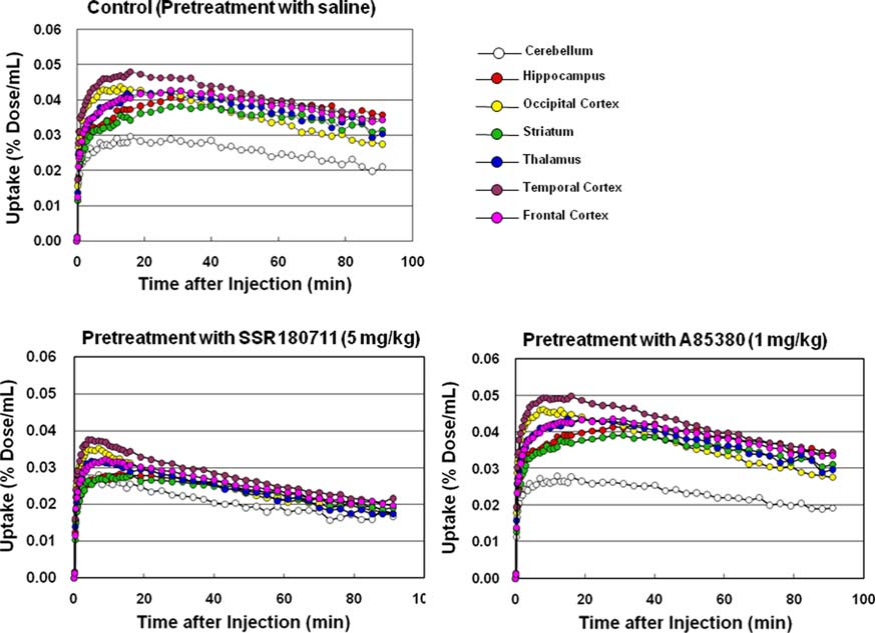
Representative time-activity curves of radioactivity (expressed as % Dose/mL) in several brain regions of a rhesus monkey after intravenous administration of [^11^C]CHIBA-1001 in control (saline pre-treated) monkey, SSR180711 (5.0 mg/kg, 30 min before)-pretreated monkey, and A85380 (1.0 mg/kg, 30 min before)-pretreated monkey.

**Figure 7:**
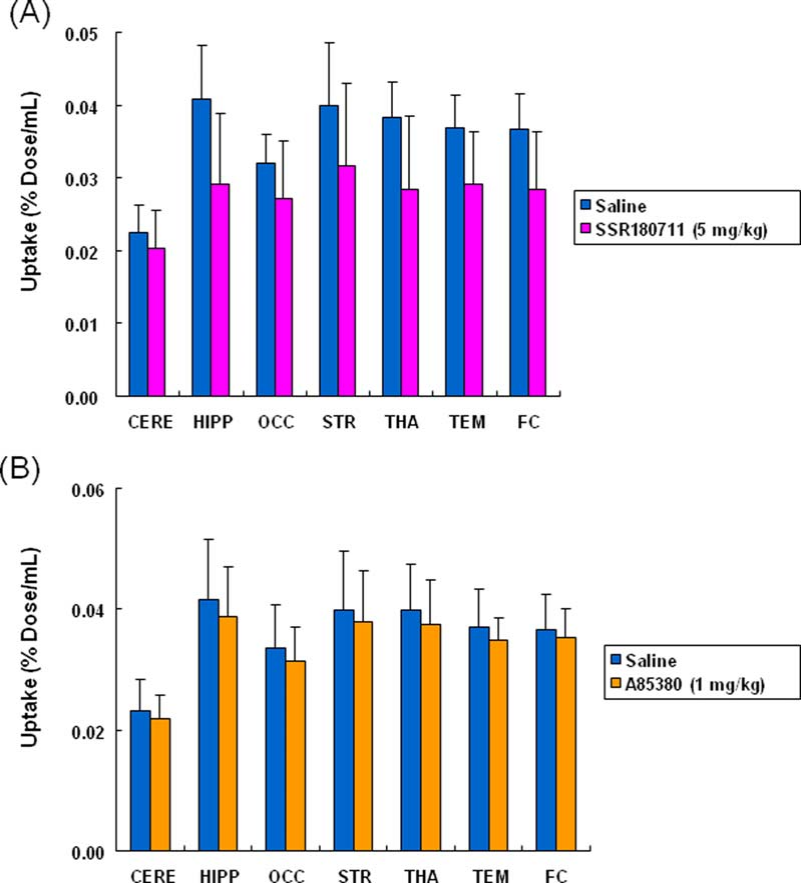
Effects of SSR180711 and A85380 on the uptake of the radioactivity in the monkey brain after intravenous administration of [^11^C]CHIBA-1001. (A): Uptake values (expressed as % Dose/mL) of [^11^C]CHIBA-1001 in several brain regions under control (saline pre-treated) group (during 70–91 min post-injection) and SSR180711 (5.0 mg/kg, 30 min before) treated groups. Data were the mean±S.D. of three monkeys. (B): Uptake values (expressed as % Dose/mL) of [^11^C]CHIBA-1001 in several brain regions under control (saline pre-treated) group (during 70–91 min post-injection) and A85380 (1.0 mg/kg, 30 min before) treated groups. Data were the mean±S.D. of three monkeys. CERE: cerebellum, HIPP: hippocampus, OCC: occipital cortex, STR: striatum, THA: thalamus, TEM: temporal cortex, FC: frontal cortex

Baseline PET scans showed rapid brain penetration and accumulation of [^11^C]CHIBA-1001 in the monkey brain (Figures 5–7). The peak time of radioactivity in the other brain regions (occipital cortex, temporal cortex, frontal cortex, striatum, thalamus, and cerebellum) was about 10 min after administration of [^11^C]CHIBA-1001, whereas the peak time of radioactivity in the hippocampus was about 30 min after administration. The distribution of radioactivity in the striatum, thalamus, hippocampus, occipital cortex, temporal cortex, and frontal cortex 40–60 min after administration of the radioligand was higher than that in the cerebellum, consistent with the distribution of α7 nAChRs in the monkey brain [25]–[27]. Uptake of radioactivity (during 70–91 min) in the brain regions except the cerebellum (low receptor density) after intravenous administration of [^11^C]CHIBA-1001 was decreased by pretreatment with SSR180711 (5.0 mg/kg) although these differences failed to reach statistical significance because of small number (n = 3) of monkey (Figures 7A). Furthermore, a preliminary study indicated that the uptake of radioactivity in the brain regions after intravenous administration of [^11^C]CHIBA-1001 was also decreased by pretreatment with another α7 nAChR agonist A844606 (5.0 mg/kg, i.v., 30 min before) [30] (Supplemental Figure S1). However, the uptake of radioactivity in the brain regions after intravenous administration of [^11^C]CHIBA-1001 was not altered by pretreatment with the selective α4β2 nAChR agonist A85380 (1.0 mg/kg, i.v., 30 min)[28], [29](Figures 5, 6, and 7B).

In the described in discussion section, it is likely that [^11^C]CHIBA-1001 is superior to [^76^Br]SSR180711 because of high brain uptake and lower half-life of [^11^C]. Therefore, [^11^C]CHIBA-1001 was used in the subsequent studies.

### Phencyclidine (PCP)-treated monkeys

**Figure 8:**
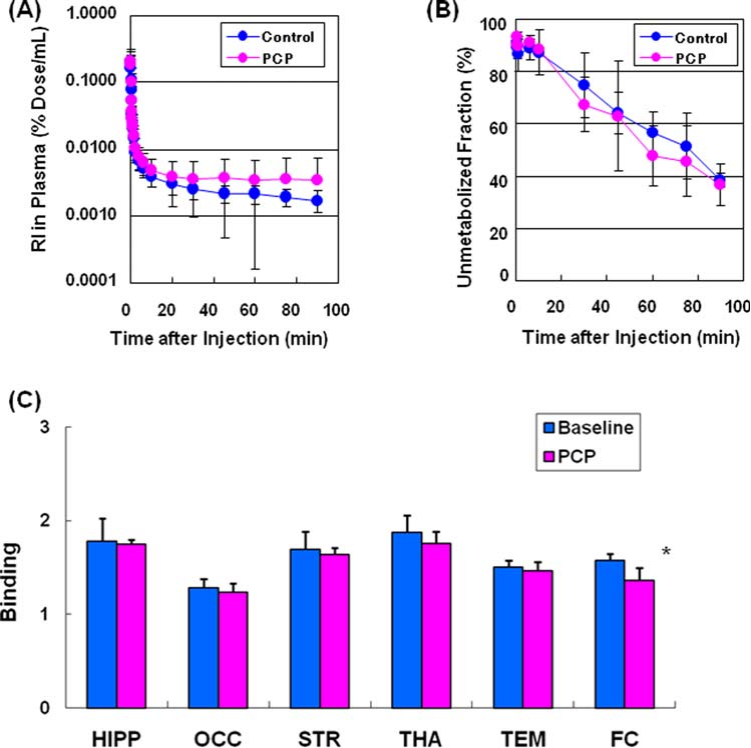
Effects of subchronic administration of PCP on the binding in monkey brain after intravenous administration of [^11^C]CHIBA-1001. (A): Radioactivity in the plasma of control (baseline; n = 4) and PCP-treated (n = 4) groups after intravenous administration of [^11^C]CHIBA-1001. Data were the mean±S.D. of four monkeys. (B): Percentage of unmetabolized fraction in the plasma of control and PCP-treated groups after intravenous administration of [^11^C]CHIBA-1001. Data were the mean±S.D. of four monkeys. (C): Receptor binding in the several brain regions of control and PCP-treated groups. Data were the mean±S.D. of four monkeys. *p<0.05 as compared to control (baseline) group (Paired t-test).

The N-methyl-D-aspartate (NMDA) receptor antagonist phencyclidine (PCP) has been used as an animal model of schizophrenia, since it has been shown to cause schizophrenia-like symptoms in humans [31]–[35]. We performed two PET scans, one before (baseline) and one 1-day after subchronic administration of PCP (0.3 mg/kg, twice a day for 13 days). Subchronic administration of PCP did not alter the time-curve of the radioactivity or the percentage of unmetabolized fraction in the plasma of monkeys (Figure 8). Interestingly, subchronic administration of PCP decreased the binding of [^11^C]CHIBA-1001 in several regions (frontal cortex, temporal cortex, occipital cortex, striatum, thalamus, and hippocampus) of the monkey brain; the difference of binding in the frontal cortex was statistically significant (t = 5.73, df = 3, p = 0.011) between the two groups (Figure 8C), consistent with a previous report using mice [35].

## Discussion

In the present study, we have developed two PET ligands, [^76^Br]SSR180711 and [^11^C]CHIBA-1001. It is likely that [^11^C]CHIBA-1001 is superior to [^76^Br]SSR180711 for the following reasons. First, [^11^C]CHIBA-1001 can be synthesized using an in house cyclotron, whereas [^76^Br]SSR180711 cannot. Second, the radiation exposure dose in humans by [^11^C]CHIBA-1001 PET study is lower than that of [^76^Br]SSR180711 because of the short half-life (the half-lives of [^11^C] and [^76^Br] are 20.4 min and 16.2 hours, respectively). Third, the short half life allows several repetitions of [^11^C]CHIBA-1001 PET in a single day. Fourth, brain uptake of [^11^C]CHIBA-1001 is higher than that of [^76^Br]SSR180711.

We have demonstrated that [^11^C]CHIBA-1001 is a novel PET ligands for *in vivo* imaging of α7 nAChRs in the non-human primate brain. First, an *in vitro* receptor binding study showed that CHIBA-1001 is a highly selective ligand at α7 nAChRs, since this ligand was found to be devoid of activity for the standard receptor binding profile. Second, an *in vivo* PET study using conscious monkeys demonstrated a high accumulation into the brain after intravenous administration of [^11^C]CHIBA-1001. The regional distribution of radioactivity in the monkey brain after intravenous administration of [^11^C]CHIBA-1001 is consistent with the distribution of α7 nAChRs in the monkey brain [25]–[27]. Furthermore, the uptake of radioactivity in the monkey brain regions was blocked by pretreatment with the selective α7 nAChR agonist SSR180711 and A844606, but not the selective α4β2 nAChR agonist A85380. Third, we found a reduction of [^11^C]CHIBA-1001 binding in the frontal cortex of the monkey brain after subchronic administration of PCP.

Recently, we reported that the repeated administration of PCP (10 mg/kg/day for 10 days) significantly decreased the density of α7 nAChRs in the frontal cortex of the mouse brain [35], consistent with our monkey data. The precise mechanism(s) underlying how repeated PCP administration could modulate α7 nAChRs in the brain are currently unknown. It has been reported that the immunoreactivity of α7 nAChRs in the prefrontal cortex of schizophrenics was significantly decreased compared to that in normal controls [36]. Interestingly, α7 nAChR agonists can increase the release of glutamate from the presynaptic terminals, resulting in stimulation of the NMDA receptors on the postsynaptic neurons, suggesting that stimulation at α7 nAChRs may potentiate the NMDA receptors [7], [37], [38]. Taken together, these findings suggest that α7 nAChRs may interact with the NMDA receptors in the brain, although further study on the cross-talk between α7 nAChRs and NMDA receptors in the brain is necessary [7], [37], [38].

A postmortem human brain study demonstrated decreased expression of hippocampal α7 nAChRs in schizophrenic patients [12], suggesting that schizophrenic patients have fewer α7 nAChRs in the hippocampus, a condition which may lead to the failure of cholinergic activation of the inhibitory interneurons, manifesting clinically as decreased gating of responses to sensory stimulation [12]. Deficient inhibitory processing of the P50 auditory evoked potential is a pathophysiological feature of schizophrenia [3], [39]–[41] and Alzheimer's disease [42], and it has been suggested that α7 nAChRs play a critical role in this phenomenon [40], [41], [43], [44]. In the present study, using [^11^C]CHIBA-1001 and PET, we could detect the reduction of α7 nAChRs in the frontal cortex in a non-human primate PCP model of schizophrenia although semi-quantitative analysis using Logan plot analysis was performed in this study. Taken together, these results suggest that it would be of great interest to examine whether α7 nAChRs are altered in the intact brain of patients with schizophrenia and Alzheimer's disease by using [^11^C]CHIBA-1001 and PET.

Based on the above findings, α7 nAChRs are the most attractive target for potential therapeutic drugs in several neuropsychiatric diseases [3]–[11], [43], [44]. A number of pharmaceutical industries have developed selective α7 nAChR agonists for the treatment of neuropsychiatric diseases, including schizophrenia and Alzheimer's disease, and clinical trials of some drugs have been started. Using [^11^C]CHIBA-1001 and PET, it will be possible to measure the relationship between the receptor occupancy and the dose of α7 nAChR agonists in the human brain, since this radioligand can be used for quantitative occupancy assessment of α7 nAChRs.

In conclusion, the present study presents the successful *in vivo* characterization of α7 nAChRs in the conscious monkey brain using [^11^C]CHIBA-1001 and PET. Therefore, *in vivo* PET imaging of α7 nAChRs in the intact human brain provides a method for quantitative study of α7 nAChR-related pathophysiology in neuropsychiatric diseases. In addition, the *in vivo* determination of receptor occupancy allows for the demonstration of target engagement and assessment of titration for potential dose regimens. A clinical PET study in healthy human subjects using [^11^C]CHIBA-1001 is currently underway.

## Materials and Methods

### Synthesis of the precursor and CHIBA-1001

SSR180711, CHIBA-1001 and the precursor, 4-(tributylstannyl)phenyl 2,5- diazabicyclo[3.2.2]nonane -2-carboxylate (Figure 1), were synthesized as described in the Supplemental Method S1.

### [^125^I]α-Bungarotoxin binding

The binding assay using [^125^I]α-bungarotoxin was performed as described in a previous report [45] with a slight modification (See Supplemental Method S2).

### Synthesis of [^75^Br]SSR180711 and [^11^C]CHIBA-1001

[^76^Br]SSR180711 and [^11^C]CHIBA-1001 were synthesized by bromination and methylation of the precursor, respectively (See Supplemental Method S3).

### Subjects

Eleven young-adult male rhesus monkeys (*Macaca mulatta*) weighing from 4 to 6 kg were used for PET measurements. The monkeys were maintained and handled in accordance with the recommendations of the US National Institutes of Health and also the guidelines of the Central Research Laboratory, Hamamatsu Photonics (Hamamatsu, Shizuoka, Japan). The animal experimental procedure was approved by the Animal Care and Use Committee of Hamamatsu Photonics and Chiba University. Over the course of 3 months, the monkeys were trained to sit on a chair twice a week. The magnetic resonance images (MRI) of all monkeys were obtained with a Toshiba MRT-50A/II (0.5T) under anesthesia with pentobarbital. The stereotactic coordinates of PET and MRI were adjusted based on the orbitomeatal (OM) line with monkeys secured in a specially designed head holder [46]. At least 1 month before the PET study, an acrylic plate, with which the monkey was fixed to the monkey chair, was attached to the head under pentobarbital anesthesia as described previously [47].

### PET scans

PET data were collected on a high-resolution PET scanner (SHR-7700; Hamamatsu Photonics K.K., Hamamatsu, Japan) with a transaxial resolution of 2.6-mm full-width at half-maximum (FWHM) and a center-to-center distance of 3.6 mm [48]. The PET camera allowed 31 slices for imaging to be recorded simultaneously. After an overnight fast, animals were fixed to the monkey chair with stereotactic coordinates aligned parallel to the OM line. A cannula was implanted in the posterior tibial vein of the monkey for administration of [^76^Br]SSR180711 or [^11^C]CHIBA-1001. [^76^Br]SSR180711 or [^11^C]CHIBA-1001 was injected through the posterior tibial vein cannula 30 min after administration of saline (control), SSR180711 (5.0 mg/kg, i.v.), or A85380 (1.0 mg/kg, i.v.; Sigma-Aldrich Co., Ltd., St Louis, MO). PET images were acquired over 91 min (10 s×6 frames, 30 s×6 frames, 1 min×12 frames, and 3 min×25 frames). Summation images from 70 to 91 min postinjection were constructed. PET scans were reconstructed using filtered backprojection in a 100×100 matrix, with a voxel size of 1.2 mm×1.2 mm×3.6 mm. Each MRI was coregistered to a summation image. Due to the very short half-life of ^11^C (20.4 min), a time lag of at least 3 hr between scans provided sufficient decay time of radioactivity in monkeys (approximately 1/400 of the injected dose). Therefore, the level of radioactivity associated with the previous injection of labeled compound would not interfere with the next scan as previously reported [49], [50].

Next, we examined the effects of subchronic administration of the NMDA receptor antagonist phencyclidine (PCP: 0.3 mg/kg, i.m., twice a day for 13 days) on the distribution of [^11^C]CHIBA-1001 binding in the monkey brain. In the control (n = 4), PET scans were performed before PCP administration. One day after subchronic administration of PCP, PET scans were performed as described above.

To assess the semi-quantitative analysis of PET data, arterial samples were obtained every 8 s from injection to 64 s, and then again at 1.5, 2.5, 4, 6, 10, 20, 30, 45, 60, and 90 min after [^11^C]CHIBA-1001 injection. Blood samples of [^11^C]CHIBA-1001 were centrifuged to separate the plasma, weighed, and subjected to radioactivity measurement. For metabolite analysis, methanol was added to some plasma samples, the resulting solutions were centrifuged, and the supernatants were developed with a thin-layer chromatography (TLC) plate (AL SIL G/UV; Whatman, Kent, UK) using a mobile phase of dichloromethane:diethyl ether:ethanol:triethylamine (20:20:2:2). At each sampling time point for analysis, the ratio of radioactivity in the unmetabolized fraction to that in the total plasma (metabolite plus unmetabolite) was determined using a phosphoimaging plate (BAS-1500 MAC; Fuji Film Co., Tokyo, Japan). The metabolite-corrected plasma curve was obtained.

### Kinetic analysis

Time-activity curves of radioactivity in each region of interest (ROI) in the brain and metabolite-corrected arterial plasma were determined. Analysis of the Logan plot provides the linear function of the free receptor concentration, which is known as the distribution volume [51]. In reversibly labeled compounds, the Logan plot becomes linear after a certain period of time with a slope (*K*) that is equal to the steady-state distribution volume. In the preliminary semi-quantitative analysis, the ratios of *K* in each ROI (*K* (ROI)) to *K* in the cerebellum (*K* (CE)) were calculated to determine the binding of α7 nAChRs in the monkey brain.

### Statistical analysis

Statistical analysis of the control (baseline) and drug (SSR180711 or A85380) -treated groups was performed by paired t-test. Statistical analysis of the control (baseline) and PCP-treated groups was also performed by paired t-test. Significance was set at p<0.05.

## Supporting Information

Figure S1 Effects of the another alpha7 nAChR agonist A844606 on the uptake of the radioactivity in the monkey brain after intravenous administration of [11C]CHIBA-1001. Representative time-activity curves of radioactivity (expressed as % Dose/mL) in several brain regions of a rhesus monkey after intravenous administration of [11C]CHIBA-1001 in control (saline pre-treated) monkey, and A844606 (1.0 mg/kg, 30 min before)-pretreated monkey.
(0.14 MB TIF)

Method S1 Preparation of SSR180711, CHIBA-1001 and precursor.
(0.08 MB DOC)

Method S2 [125I]alpha-Bungarotoxin binding
(0.03 MB DOC)

Method S3 Synthesis of [75Br]SSR180711 and [11C]CHIBA-1001
(0.03 MB DOC)

Table S1 Inhibition effect of CHIBA-1001 (10 uM) on radioligand binding to various receptors
(0.07 MB DOC)

Table S2 Inhibition effect of CHIBA-1001 (1 µM) on radioligand binding to various receptors
(0.04 MB DOC)
