# Supplementary figures and images for: [11C]CHIBA-1001 as a Novel PET Ligand for α7 Nicotinic Receptors in the Brain: A PET Study in Conscious Monkeys
Source: PLoS One. 2008 Sep 18;3(9):e3231. doi: 10.1371/journal.pone.0003231 (PMC2529405; doi:10.1371/journal.pone.0003231)

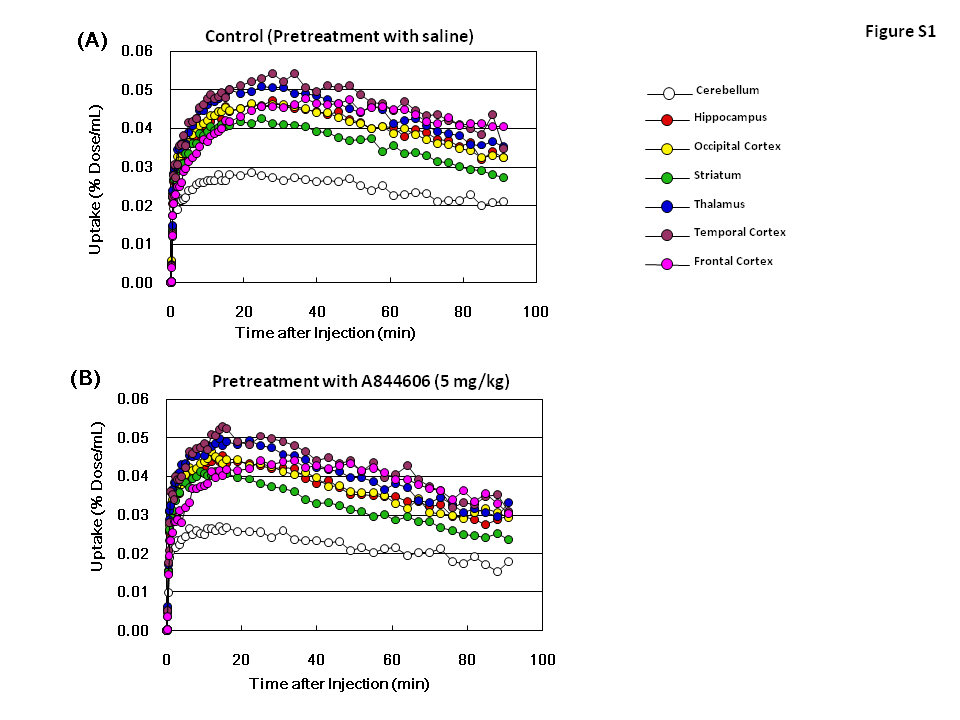

Supplement: Figure S1 — Effects of the another alpha7 nAChR agonist A844606 on the uptake of the radioactivity in the monkey brain after intravenous administration of [11C]CHIBA-1001. Representative time-activity curves of radioactivity (expressed as % Dose/mL) in several brain regions of a rhesus monkey after intravenous administration of [11C]CHIBA-1001 in control (saline pre-treated) monkey, and A844606 (1.0 mg/kg, 30 min before)-pretreated monkey. (0.14 MB TIF) [file pone.0003231.s001.tif]
