# Supplementary material for: [11C]CHIBA-1001 as a Novel PET Ligand for α7 Nicotinic Receptors in the Brain: A PET Study in Conscious Monkeys
Source: PLoS One. 2008 Sep 18;3(9):e3231. doi: 10.1371/journal.pone.0003231 (PMC2529405; doi:10.1371/journal.pone.0003231)
Supplement: Method S1 — Preparation of SSR180711, CHIBA-1001 and precursor. (0.08 MB DOC) [file pone.0003231.s002.doc]

**Supplemental Method S1**

The precursor (compound 104) for preparation of the radioactive ligand, [^11^C]CHIBA-1001, was obtained through 4 steps synthesis (ref-1, ref-2) from commercially available quinuclidone hydrochloride. The lactam (compound 101) was given by Schmidt reaction of the starting quinuclidone hydrochloride in very low yield. Reduction of the lactam with lithium aluminum hydride afforded the secondary amine (compound 102), which was led in moderate yield to the carbamate (compound 103: SSR180711) using 4-bromophenyl chloroformate. The bromine atom in compound 103 was converted in moderate yield to the tributylstannyl function according to the well known method, i.e., reaction of compound 103 with bis(tributyltin) and tetrakis(triphenylphosphine)palladium (0) as a catalyst in refluxing toluene.

The cold substance of CHIBA-1001 (compound 105) was synthesized from the secondary amine (compound 102) using 4-methylphenyl chloroformate in moderate yield.

**Synthesis of the precursor, 4-(tributylstannyl)phenyl 2,5- diazabicyclo[3.2.2]nonane -2-carboxylate (compound 104)**

**3-Oxo-1,4-diazabicyclo[3.2.2]nonane (compound 101）^1)^**

Quinuclidone hydrochloridde (25 g, 0.155 mol) was dissolved in concentrated sulfuric acid (62.5mL) and the solution was cooled to 0-10°C. Sodium azide (25 g, 0.385 mol) was added to the solution at 0-10℃, and the resulting mixture was stirred at 5℃ for 4 hours. The reaction mixture was diluted with water (125 mL) and quenched with 15 N sodium hydroxide. After the quench, pH of the reaction mixture was approximately 14. Sodium sulfate formed was filtered off and the filtrate was extracted three times with chloroform. The combined extracts were dried over anhydrous magnesium sulfate and concentrated. This residual solid was chromatographed over silica gel (Chloroform: Methanol= 9:1) to give 1.76 g of compound 101. (yield 8%)

^１^H NMR (CDCl_3_) δ7.24(br s, 1H, CONH), 3.70 (S, 2H), 3.52-3.38 (m, 1H), 3.24-2.96 (m, 4H), 2.26-2.05 (m, 2H), 2.01-1.77 (m, 2H)

**1,4-diazabicyclo[3.2.2]nonane (compound 102)^1)^**

Lithium aluminum hydride (0.94 g, 0.025 mol) was slurried in dry tetrahydrofuran (120 mL) and compound 101 (1.7 g, 0.012 mol) was added at room temperature. The reaction mixture was then refluxed for overnight. The mixture was cooled to room temperature and quenched with water (1.2 mL). The resulting salts were filtered off and washed several times with tetrahydrofuran. These washings and the filtrate were combined, dried over anhydrous magnesium sulfate and concentrated to give 1.45 g of compound 102. (yield 95%)

^１^H NMR (CDCL_3_) δ3.38-3.24 (m, 1H), 3.16-2.90 (m, 8H), 2.19-1.54 (m, 5H)

**4-bromophenyl 2,5-diazabicyclo[3.2.2]nonane-2-carboxylate (compound 103: SSR180711)^2)^**

4-Bromophenyl chloroformate (2.96 g, 0.013 mol) was added to a mixture of compound 102 (1.45 g, 0.011 mol), 4-dimethylaminopyridine (1.4 g, 0.011 mol), pyridine (1.9 mL), and dichloromethane (38.4 mL) at -10°C. Cooling bath was removed after the addition and the mixture was allowed to stir at room temperature for 15 hours. The mixture was diluted with dichloromethane (40 mL), and the organic layer was washed with saturated sodium hydrogencarbonate aqueous solution, with water, and then with brine. The organic layer was dried over anhydrous magnesium sulfate and concentrated to give crude residual oil. The residual oil was chromatographed over amine coated silicagel (Chloroform: Methanol= 200:1) and then purified with preparative GPC (JAIGEL 1H+2Hφ20×600mm, Chloroform) to give 1.87 g of compound 103. (yield 50 %)

^１^H NMR (CDCl_3_) δ7.56-7.38(d, 2H, aromatic), 7.10-6.94 (dd, 2H, aromatic), 4.50-4.28 (m, 1H), 3.88-3.64 (m, 2H), 3.24-2.90 (m, 6H), 2.22-1.94 (m, 2H), 1.90-1.60 (m, 2H)

**4-(Tributylstannyl)phenyl 2,5-diazabicyclo [3.2.2] nonane-2-carboxylate (compound 104)**

To a solution of compound 103 (1.0 g, 0.003 mol) in dry toluene (30 mL) was added tetrakis(triphenylphosphine)palladium (0) (0.18 g, 0.00015 mol). Bis(tributyltin) (4.45 g 0.0077 mol) was added, and the reaction mixture was then refluxed for 7 hours. The mixture was cooled to room temperature and stirred overnight. The reaction mixture was filtered and washed with toluene. These washings and the filtrate were combined, and concentrated to give 5.87 g of crude residual oil. The residual oil was chromatographed over silicagel (n-Hexane: EtOAc= 3:2) to give 0.7 g of compound 104. (yield 43%)

^１^H NMR (CDCl_3_) δ7.56-7.31 (d, 2H, aromatic), 7.14-6.91 (dd, 2H, aromatic), 4.51-4.24 (m, 1H), 3.89-3.55 (m, 2H), 3.30-2.90 (m, 6H), 2.24-1.94 (m, 2H), 1.88-0.75 (m, 29H)

**Synthesis of CHIBA-1001, 4-Methylphenyl 2,5-diazabicyclo[3.2.2]nonane-2-carboxylate (compound 105) ^2)^**

4-Methylphenyl chloroformate (0.65 g, 0.0038 mol) was added to a mixture of compound 102 (0.44 g, 0.0035 mol), 4-dimethylaminopyridine (0.43 g, 0.0035 mol), pyridine (0.58 mL), and dichloromethane (12 mL) at -5°C. Cooling bath was removed after the addition and the mixture was allowed to stir at room temperature for 15 hours. The mixture was diluted with dichloromethane (30 mL), and the organic layer was washed with saturated sodium hydrogencarbonate aqueous solution, with water, and then with brine. The organic layer was dried over anhydrous magnesium sulfate and concentrated to give crude residual solid. The residual solid was chromatographed over amine coated silicagel (Chloroform) and then purified with preparative GPC (JAIGEL 1H+2Hφ 20×600mm, Chloroform) to give 0.38 g of the compound 105. (yield 42%)

^１^H NMR (CDCl_3_) δ7.21-7.10 (d, 2H, aromatic), 7.06-6.94 (dd, 2H, aromatic), 4.49-4.32 (m, 1H), 3.87-3.70 (m, 2H), 3.21-2.93 (m, 6H), 2.33 (s, 3H, CH_3_), 2.18-1.99 (m, 2H), 1.85-1.64 (m, 2H)

**References**

1. EP0215650 (Jefson, Martin Raymond et al, Pfizer Inc. Date of publication 12.09.1986)
2. EP1231212 (O’Donnell et al, Pfizer Products Inc. Date of publication 14.08.2002)
