# Supplementary material for: [11C]CHIBA-1001 as a Novel PET Ligand for α7 Nicotinic Receptors in the Brain: A PET Study in Conscious Monkeys
Source: PLoS One. 2008 Sep 18;3(9):e3231. doi: 10.1371/journal.pone.0003231 (PMC2529405; doi:10.1371/journal.pone.0003231)
Supplement: Method S2 — [125I]alpha-Bungarotoxin binding (0.03 MB DOC) [file pone.0003231.s003.doc]

**Supplemental Method S2**

**[^125^I]α-Bungarotoxin binding**

Binding assay was performed by the previous report (45) with a slight modification. Brain (exclude cerebellum and pons-medulla) from male SD rat (Japan Crea Ltd., Hamamatsu, Japan) was homogenized in 15 volumes of 0.32 M sucrose and centrifuged at 1,000 x g for 10 min (4°C). The supernatant was centrifuged at 20,000 x g for 20 min (4°C). The resultant P_2_ pellets were homogenized with a Polytron in ice-cold distilled water and spun at 8,000 x g for 20 min (4°C). The supernatant and loose buffy coat were carefully removed and centrifuged at 38,000 x g for 20 min (4°C). The membrane pellet was washed with ice-cold distilled water and recentrifuged at 38,000 x g for 20 min (4°C) before storage at -80°C. The final pellet was suspended in the buffer (118 mM NaCl, 4.8 mM KCl, 2.5 mM CaCl_2_, 1.2 mM MgSO_4_, 20 mM Na-HEPES, pH 7.5 at RT) for receptor binding assay. Aliquots of membrane suspension (200 μl) were added to reaction mixture containing 0.5 nM [^125^I]α-bungarotoxin (GE Bioscience, U.K.) and the indicated concentrations of test drugs in a final volume of 0.5 ml. Non-specific binding was estimated in the presence of 1 mM (-)-nicotine. Binding was conducted at 37°C for 3 hours. Bound radioactivity was isolated by rapid vacuum filtration onto Whatman GF/B filters pretreated with 0.5 % PEI including 0.1 % BSA for 3 hours.
