# Supplementary material for: [11C]CHIBA-1001 as a Novel PET Ligand for α7 Nicotinic Receptors in the Brain: A PET Study in Conscious Monkeys
Source: PLoS One. 2008 Sep 18;3(9):e3231. doi: 10.1371/journal.pone.0003231 (PMC2529405; doi:10.1371/journal.pone.0003231)
Supplement: Method S3 — Synthesis of [75Br]SSR180711 and [11C]CHIBA-1001 (0.03 MB DOC) [file pone.0003231.s004.doc]

**Supplemental Method S3**

**Synthesis of [^75^Br]SSR180711:** [^76^Br]SSR180711 was synthesized by the method of chloramine-T with the precursor and [^76^Br]bromide (Sumitomo Heavy Industries, Ltd., Examination & Inspection Ehime, Japan)(Fig. 1). [^76^Br]bromide ethanol solution was heated at 70°C under argon gas flow and concentrated to 200 μL. The precursor (1.0 mg) was dissolved in the 250 μL of 1% of acetic acid ethanol solution. The precursor ethanol solution (250 μL) and 200 mM chloramine-T ethanol solution (25 μL) were added to 200 μL of [^76^Br]bromide ethanol solution. Reactant was heated at 75°C for 30 min. The mixture of reaction solution was transferred to the preparative HPLC system, using a Megapak SIL C18-10 column (7.6 mm in inner diameter x 250 mm in length, Jasco, Tokyo, Japan). The radioactive peak fraction eluted by CH_3_CN / (1000 mL of 30 mM CH_3_COONH_4_ + 4 mL of CH_3_COOH) = 600 / 400 at a flow rate of 6 ml/min was collected into an evaporation flask, evaporated and the residue was re-dissolved with 3 ml of saline, the solution was passed through a 0.22-μm pore size filter before intravenous administration to the monkey. Chemical and radiochemical analyses of [^76^Br]SSR180711 was performed by HPLC in a system consisting of a column (Finepak SIL C18-S, 4.6 mm in inner diameter x 150 mm in length, Jasco, Tokyo, Japan), using CH_3_CN / 30mM CH_3_COONH_4_ / CH_3_COOH (500 / 500 / 2) as a mobile phase at a flow rate of 2 ml/min

**Synthesis of [^11^C]CHIBA-1001:** Carbon-11 (^11^C) was produced by bombardment of N_2_ gas with 18 MeV proton at 20 μA current for 60 min using a cyclotron (HM-18, Sumitomo Heavy Industry, Tokyo, Japan) at Hamamatsu Photonics PET center and obtained as [^11^C]CO_2_.

[^11^C]CHIBA-1001 was synthesized by *N*-methylation of the precursor with [^11^C]methyl iodide (**Figure 1**). [^11^C]CO_2_ was transferred under N_2_ flow (400ml/min) from a target chamber into the reaction vessel containing 0.5 ml of 0.1 M LiAlH_4_/THF cooled at –15°C with a compressed air blower. Immediately after carboxylation, the reaction vessel was heated to evaporate THF at 120°C under a N_2_ flow of 400 ml/min. After removal of THF, 0.5 ml of HI was added. The solution was heated at 120°C for 1 - 2 min under N_2_ flow to transfer the [^11^C]methyl iodide into the another reaction vessel, which contained tris(dibenzylideneaceton)dipalladium (4.6 mg) and tri-o-tolylphosphine (6.2 mg) dissolved in the 0.3 ml of DMF. After [^11^C]methyl iodide was collected, the solution of containing the precursor (4 mg), copper (I) chloride (1.0 mg) and potassium carbonate (1.4 mg) in DMF (0.3 ml) was transferred to the vessel. The reaction vessel was heated at 70°C for 5 min. The mixture of reaction solution was transferred to the HPLC injection unit through the glass filter to remove solid reagents, and injected into a preparative HPLC system, using a Megapak SIL C18-10 column (7.6 mm in inner diameter x 250 mm in length, Jasco, Tokyo, Japan). The radioactive peak fraction eluted by CH_3_CN / 30 mM CH_3_COONH_4_ / CH_3_COOH (600 / 400 / 2) at a flow rate of 6 ml/min was collected into an evaporation flask, evaporated and the residue was re-dissolved with 3 ml of saline, the solution was passed through a 0.22-μm pore size filter before intravenous administration to the monkey.

Chemical and radiochemical analyses of [^11^C]CHIBA-1001 was performed by HPLC in a system consisting of a column (Finepak SIL C18-S, 4.6 mm in inner diameter x 150 mm in length, Jasco, Tokyo, Japan), using CH_3_CN / 30mM CH_3_COONH_4_ / CH_3_COOH (500 / 500 / 2) as a mobile phase at a flow rate of 2 ml/min
