# Supplementary material for: [11C]CHIBA-1001 as a Novel PET Ligand for α7 Nicotinic Receptors in the Brain: A PET Study in Conscious Monkeys
Source: PLoS One. 2008 Sep 18;3(9):e3231. doi: 10.1371/journal.pone.0003231 (PMC2529405; doi:10.1371/journal.pone.0003231)
Supplement: Table S1 — Inhibition effect of CHIBA-1001 (10 uM) on radioligand binding to various receptors (0.07 MB DOC) [file pone.0003231.s005.doc]

Supplemental Table S1. Inhibition effect of CHIBA-1001 (10 μM) on radioligand binding to various receptors

| Assay Name | Inhibition (%) | | |
| --- | --- | --- | --- |
|  | CHIBA-1001 | Positive substance Radioligand | |
| Adenosine A1 | 3.31 | 99.35 | (DPCPX) [^3^H]DPCPX |
| Adenosine A2 | 16.12 | 100.00 | (NECA) [^3^H]CGS21680 |
| α1-Adrenergic (Non-selective) | 6.44 | 99.12 | (Prazosin) [^3^H]Prazosin |
| α2-Adrenergic (Non-selective) | 1.20 | 100.00 | (Yohimbine) [^3^H]RX821002 |
| β-Adrenergic (Non-selective) | 1.01 | 100.00 | (Propranolol) [^3^H]DHA |
| Dopamine D1 | 0.32 | 97.26 | (SCH23390) [^3^H]SCH23390 |
| Dopamine D2 | 0.00 | 95.14 | ((+)-Butaclamol) [^3^H]Spiperone |
| GABA A (Agonist site) | 2.89 | 95.09 | (Muscimol) [^3^H]Muscimol |
| GABA A (BZ central) | 8.27 | 98.79 | (Diazepam) [^3^H]Flunitrazepam |
| GABA B | 0.00 | 95.52 | (GABA) [^3^H]GABA |
| Glutamate (Non-selective) | 17.01 | 100.00 | (Glutamic acid) [^3^H]Glutamic acid |
| Glutamate (AMPA) | 0.00 | 100.00 | (AMPA) [^3^H]AMPA |
| Glutamate (Kainate) | 8.78 | 100.00 | (Kainic acid) [^3^H]Kainic acid |
| Glutamate (NMDA agonist site) | 2.48 | 99.90 | (Glutamic acid) [^3^H]CGP-39653 |
| Glutamate (NMDA glycine site) | 4.89 | 96.08 | (MDL105519) [^3^H]MDL105519 |
| Glutamate (NMDA phencyclidine site) | 0.00 | 100.00 | (MK-801) [^3^H]MK-801 |
| Glycine (Strychnine sensitive) | 2.78 | 99.72 | (Strychnine) [^3^H]Strychnine |
| Histamine H1 (Central) | 2.45 | 99.49 | (Pyrilamine) [^3^H]Pyrilamine |
| Histamine H2 | 16.78 | 100.00 | (Cimetidine) [^3^H]Cimetidine |
| Histamine H3 | 72.35 | 98.77 | (α-methyl histamine) [^3^H]N-methyl histamine |
| Muscarinic M1 | 71.89 | 99.71 | (Atropine) [^3^H]Pirenzepine |
| Muscarinic M2 | 64.24 | 100.00 | (Atropine) [^3^H]AF-DX-384 |
| Nicotinic | 40.38 | 96.90 | (Nicotine) [^3^H]Nicotine |
| Opiate (Non-selective) | 6.90 | 100.00 | (Naloxone) [^3^H]Naloxone |
| Serotonin 5HT1A | 9.02 | 100.00 | (Serotonin) [^3^H]8-OH DPAT |
| Serotonin 5HT2A | 17.04 | 100.00 | (Ketanserin) [^3^H]Ketanserin |
| Serotonin 5HT3 (Human) | 56.69 | 100.00 | (MDL72222) [^3^H]GR65630 |
| Sigma (Non-selective) | 52.67 | 100.00 | (Haloperidol) [^3^H]DTG |

Test substance concentration : 10 μM, Positive substance concentration : 10 μM

Data are expressed as the mean values of duplicate samples.

The % inhibition was calculated from “100 – binding ratio”.

Binding ratio: [(B - N) / (B_0_ - N)] × 100 (%)

B: Bound radioactivity in the presence of test substance and positive substance (individual value)

B_0_: Total bound radioactivity in the absence of test substance and positive substance (mean value)

N: Non-specific bound radioactivity (mean value)
