# Supplementary material for: [11C]CHIBA-1001 as a Novel PET Ligand for α7 Nicotinic Receptors in the Brain: A PET Study in Conscious Monkeys
Source: PLoS One. 2008 Sep 18;3(9):e3231. doi: 10.1371/journal.pone.0003231 (PMC2529405; doi:10.1371/journal.pone.0003231)
Supplement: Table S2 — Inhibition effect of CHIBA-1001 (1 µM) on radioligand binding to various receptors (0.04 MB DOC) [file pone.0003231.s006.doc]

Supplemental Table S1. Inhibition effect of CHIBA-1001 (1 μM) on radioligand binding to various receptors

| Assay Name | Inhibition (%) | | |
| --- | --- | --- | --- |
|  | CHIBA-1001 | Positive substance | |
| Histamine H3 | 24.26 | 98.89 | (α-methyl histamine) |
| Muscarinic M1 | 23.36 | 98.69 | (Atropine) |
| Muscarinic M2 | 17.93 | 99.86 | (Atropine) |
| Serotonin 5HT3 (Human) | 13.63 | 97.39 | (MDL72222) |
| Sigma (Non-selective) | 13.79 | 97.58 | (Haloperidol) |

Test substance concentration : 1 μM, Positive substance concentration :1 μM

Data are expressed as the mean values of duplicate samples.

The % inhibition was calculated from “100 – binding ratio”.

Binding ratio: [(B - N) / (B_0_ - N)] × 100 (%)

B: Bound radioactivity in the presence of test substance and positive substance (individual value)

B_0_: Total bound radioactivity in the absence of test substance and positive substance (mean value)

N: Non-specific bound radioactivity (mean value)
